# Ultrasound manifestations and clinical features of nonpalpable testis in children

**DOI:** 10.1038/s41598-022-16230-2

**Published:** 2022-07-18

**Authors:** Wei Zhou, Shoulin Li, Hao Wang, Guanglun Zhou, Jianguo Wen

**Affiliations:** 1 Pediatric Urodynamic Centre, Urology, The First Affiliated Hospital of Zhengzhou University, Jianshe East Road, Zhengzhou, 450052 Henan China; 2 Department of Urology and Laboratory of Pelvic Floor Muscle Function, Shenzhen Children’s Hospital, Shenzhen, China

**Keywords:** Anatomy, Urology, Urogenital diseases, Paediatric research, Reproductive signs and symptoms

## Abstract

To explore the value of ultrasound in the preoperative diagnosis of nonpalpable testis (NPT) in children. A retrospective study of 254 cases with NPT from May 2017 to December 2021. The preoperative ultrasound examination results were compared with the surgical exploration and pathological results. There were 254 cases (312 testes) NPT has got surgery in our centre. The surgical age were from 6 month to 12 years old, the median age was 2.33 years. There were 103 cases (136 testes) diagnosed as intra-abdominal testis (IAT) by preoperative ultrasound, and 80 cases (103 testes) of extra-abdominal testis (EAT), 71 cases (73 testes) of non-viable testis (NVT). There were 102 cases (135 testes) consistented as IAT by the diagnostic laparoscopy, the preoperative ultrasound’s coincidence of IAT was 99.02%. There were 80 cases (103 testes) consistented as EAT by the diagnostic laparoscopy, the preoperative ultrasound’s coincidence rate was100%. There were 62 cases (62 testes) consistented as NVT by the diagnostic laparoscopy, there were 9 cases (11 testes) misdiagnosed, the preoperative ultrasound’s coincidence rate was 84.9%. Ultrasound can provide valuable information for the preoperative diagnosis of children with nonpalpable testicles, and especially good at identifying the EAT and IAT with normal testicular morphology.

## Introduction

Cryptorchidism is a common congenital abnormal development of the reproductive system in children, and it is one of the important causes of testicular cancer and infertility^1,2^. During physical examination, about 15–20% of children with cryptorchidism cannot touch the corresponding testis, which is called nonpalpable testis (NPT). NPT can be divided into three types: intra-abdominal testis (IAT), extra-abdominal testis (EAT) and non-viable testis (NVT)^3^. The testis of IAT and EAT are roughly normal in volume and mostly viable. IAT is mainly located near the iliac vessels, while EAT is mostly high inguinal testis. NVT includes the absence of testis, testicular remnants, which can be located in the abdominal cavity or outside the abdominal cavity. Preoperative ultrasonography can provide valuable information for the diagnosis and treatment of children with NPT, which is including the classification of NPT, the anatomical location of the testis, and the degree of testicular mobility^4,5^.

This study retrospectively analyzed the preoperative ultrasound images of 254 cases with NPT, aiming to explore the value of ultrasound in the location and qualitative diagnosis of NPT.

## Materials and methods

### Study participants

A total of 254 cases (312 testes) with NPT who underwent surgical exploration and treatment in our hospital from June 2017 to December 2021, aged from 6 months to 12 years old, the median age was 2.33 years, 112 cases were on the left side, 84 cases on the right side, and 58 cases on both sides. All cases underwent preoperative ultrasound examination.

### Instrumentation

The study utilized the MINDRAY DC-8 Color Doppler Ultrasound Diagnostic System (Mindray Company, Shenzhen, China), using a linear array probe for scanning, the frequency settings of the probe was 5–12 MHz.

### Ultrasound inspection process

All ultrasound examinations were performed by an experienced pediatric urologic sonographer in the department of urology in our hospital. All cases completed the examination in a quiet state. If the infants and young children were not actively cooperate, give milk and cartoons to help them relax for minimize the interference of the children's crying on the examination. The children is routinely placed in a supine position, and the examination site is fully exposed.

The morphological and parenchymal homogeneity scans of the testis were performed on conventional grayscale images in 3 orthogonal planes. The low-velocity flow pattern of color Doppler flow imaging (CDFI) was performed to detect testicular parenchymal vessels^6^. High color gain, low flow filter, and low velocity scale were performed to optimize detection of low velocity arterial blood flow.

**Figure 1:**
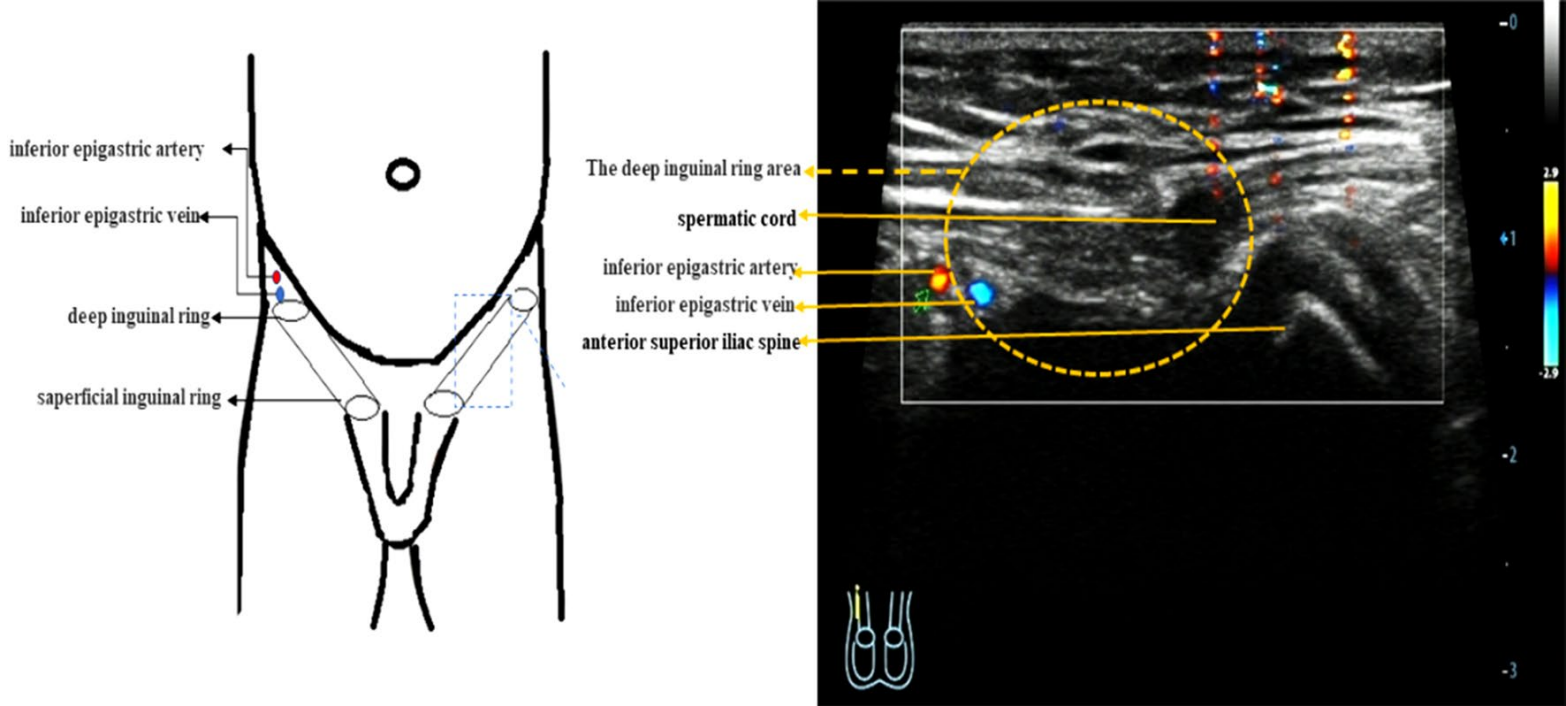
The schematic diagram and ultrasonography image of the inguinal canal in infant.

We were performed continuous dynamic scaning of the long axes and short axes of the bilateral inguinal canal and scrotum. The inferior epigastric artery could as an anatomy landmark to identify the deep inguinal ring area (Fig. 1)^7^.

If there were not showed testis in the scrotum and inguinal area, and then the inner ring of the inguinal canal, the iliac fossa and the periphery of the iliac vessels, retroperitoneum and abdominal cavity were further scanned. The position and activity of the testis, the internal echo, the size and volume, and the color Doppler blood flow were recorded and stored in the PACS system for repeat analysis.

### Diagnostic laparoscopy

All patients underwent diagnostic laparoscopy, and the results of the diagnostic laparoscopy were compared with the results of preoperative ultrasound. According to the results of diagnostic laparoscopy to decide the next treatment procedures.

### Surgical procedures

If the vas deferens and spermatic cord have entered the deep inguinal ring, further exploration of the inguinal canal was performed. Orchiopexy for high inguinal cryptorchidism. Resection for dysplastic testes and nodules at the end of the spermatic cord.

If the spermatic cord and vas deferens was blind ending in the abdominal cavity, no further surgical exploration was necessary.

If the testis was found in the abdominal cavity, the specific surgical method was determined according to the location of the testis. If the testis can be retracted to the contralateral inner ring, a one-stage orchiopexy (inguinal orchiopexy or laparoscopic orchiopexy) was performed. Fowler-Stephens orchiopexy was performed if the testis was located higher position in the abdominal cavity and cannot be retracted to the contralateral inner ring. Resection for the dysplastic testes and nodules at the end of the spermatic cord.

### Pathological examination

The testicular nubbin and remnant cord structures were removed to confirm the diagnosis by pathological examination. If the structure of seminiferous tubules was found in pathological examination, it was defined as atrophic testis, otherwise it was defined as testicular regression syndrome.

### Statistical analysis

SPSS 19.0 software was used for all statistical analyses (SPSS Inc, Chicago, IL, USA). Measurement data are expressed as mean ± standard deviation. *P* < 0.05 indicates that the difference is statistically significant. The SPSS Kappa consistency test was used to analyze the consistency of preoperative ultrasound and diagnostic laparoscopy in diagnosing three types of NPT (IAT, EAT, NVT). When the kappa value is equal to "1", it means that the two results are exactly the same. When the kappa value is equal to "− 1", the result is completely inconsistent. When the kappa value is "0", it means that the consistency of the results is random. When the kappa value is between 0.75 and 1, the consistency is good. A kappa value between 0.4 and 0.75 indicates moderate agreement. When the kappa value is between 0 and 0.4, the agreement is poor. When the kappa value is less than 0, the results are very inconsistent, and the practical significance is very small.

### Ethics approval

This retrospective review study involving human participants was in accordance with the ethical standards of the institutional and national research committee and with the 1964 Helsinki Declaration and its later amendments or comparable ethical standards. The Ethics Committee of Shenzhen Children's Hospital approved this study.

### Consent to participate

This study was performed retrospectively, and the parents of all cases gave informed consent to the surgery.

## Results

### Ultrasound features of the study participants

#### Cryptorchidism in the abdominal cavity and cryptorchidism outside the abdominal cavity (IAT and EAT)

A total of 103 cases (136 testes) of IAT were diagnosed by preoperative ultrasound, there were 33 cases on the left side, 37cases on the right side, and 33 cases on both sides. There were 102 cases (135 testes) consistented as IAT by the diagnostic laparoscopy. There were 87 cases performed laparoscopic orchidopexy, and 15 cases performed Fowler-Stephens orchidopexy. There was only one case was misdiagnosed. The preoperative ultrasound’s coincidence rate was 99.02%. The comparison list of NPT by preoperative ultrasound and diagnostic laparoscopy were shown in Table 1.

**Table 1:** The comparison list of NPT by preoperative ultrasound and diagnostic laparoscopy.

		Diagnostic laparoscopy		
		+	−	
**Preoperative ultrasound**	+	238(TP)	1(FP)	PPV = 99.58%
	−	11(FN)	62(TN)	NPV = 84.93%
		Sn = 95.58%	Sp = 98.41%	

*TP* true positive, *FP* false positive, *PPV* positive predictive value, *NPV* negative predictive value, *FN* false negative, *Sn* sensitivity, *Sp* specificity.

A total of 80 cases (103 testes) of EAT were diagnosed by preoperative ultrasound, there were 28 cases on the left side, 29cases on the right side, and 23 cases on both sides. There were 80 cases (103 testes) consistented as EAT by the diagnostic laparoscopy. There were 71 cases performed laparoscopic orchidopexy, and 6 cases performed inguinal orchidopexy, 3 cases performed Fowler-Stephens orchidopexy. The preoperative ultrasound’s coincidence rate was 100%.

**Figure 2:**
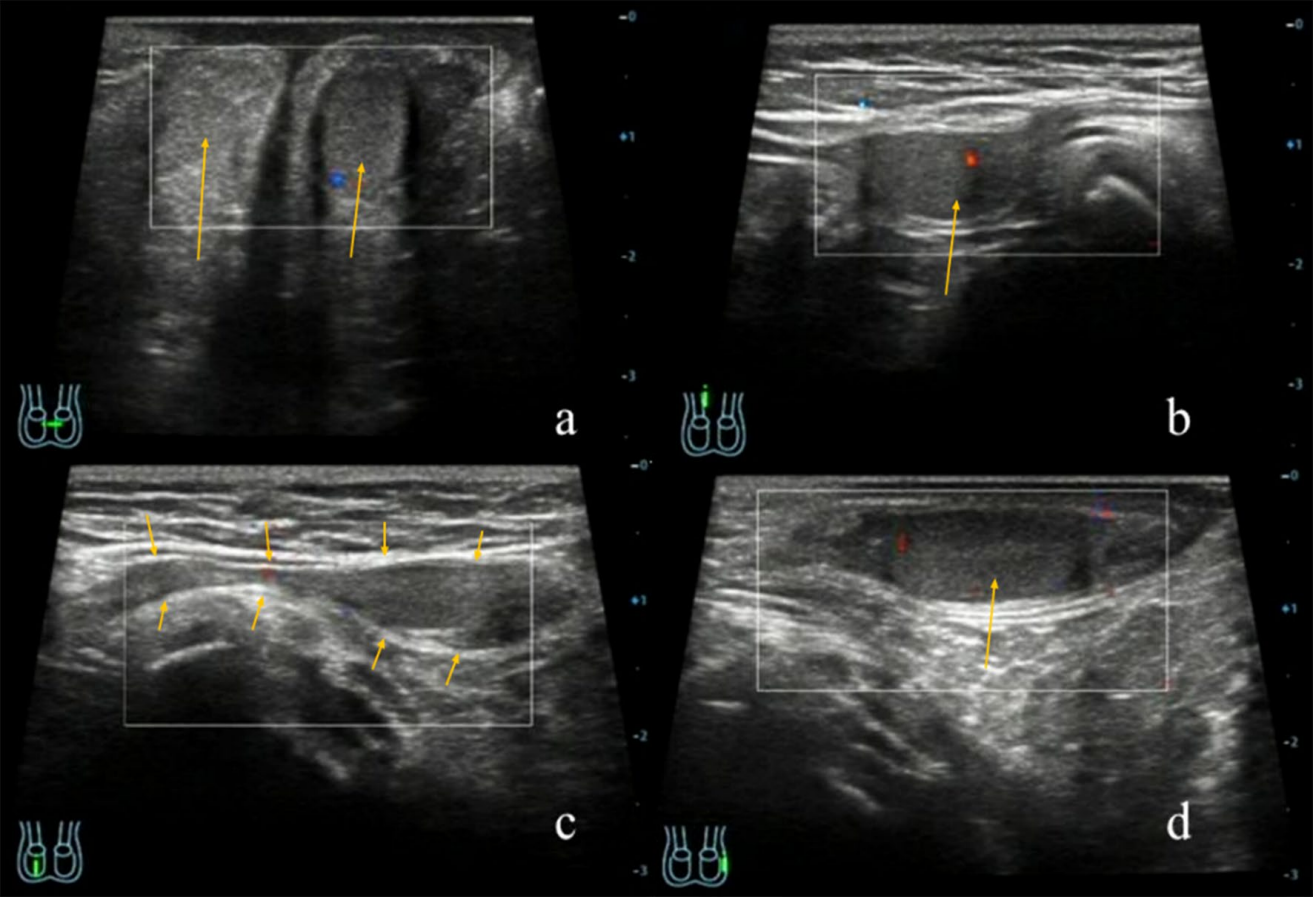
Ultrasound features of EAT on the right side of a 10-months-old boy. **(a)** The cross section of the scrotum has showed that the left testicular was in the left scrotum, and the right testicular echo was not in the right scrotum **(b)** and the upper part of the right inguinal canal near the inner ring showed the right testicular, and CDFI showed punctate blood flow signals inside **(c)** and the right testis can be moved down to the middle part of the right inguinal canal **(d)** and the CDFI of the left testis longitudinal section showed punctate blood flow signal in the left testis (arrows).

**Figure 3:**
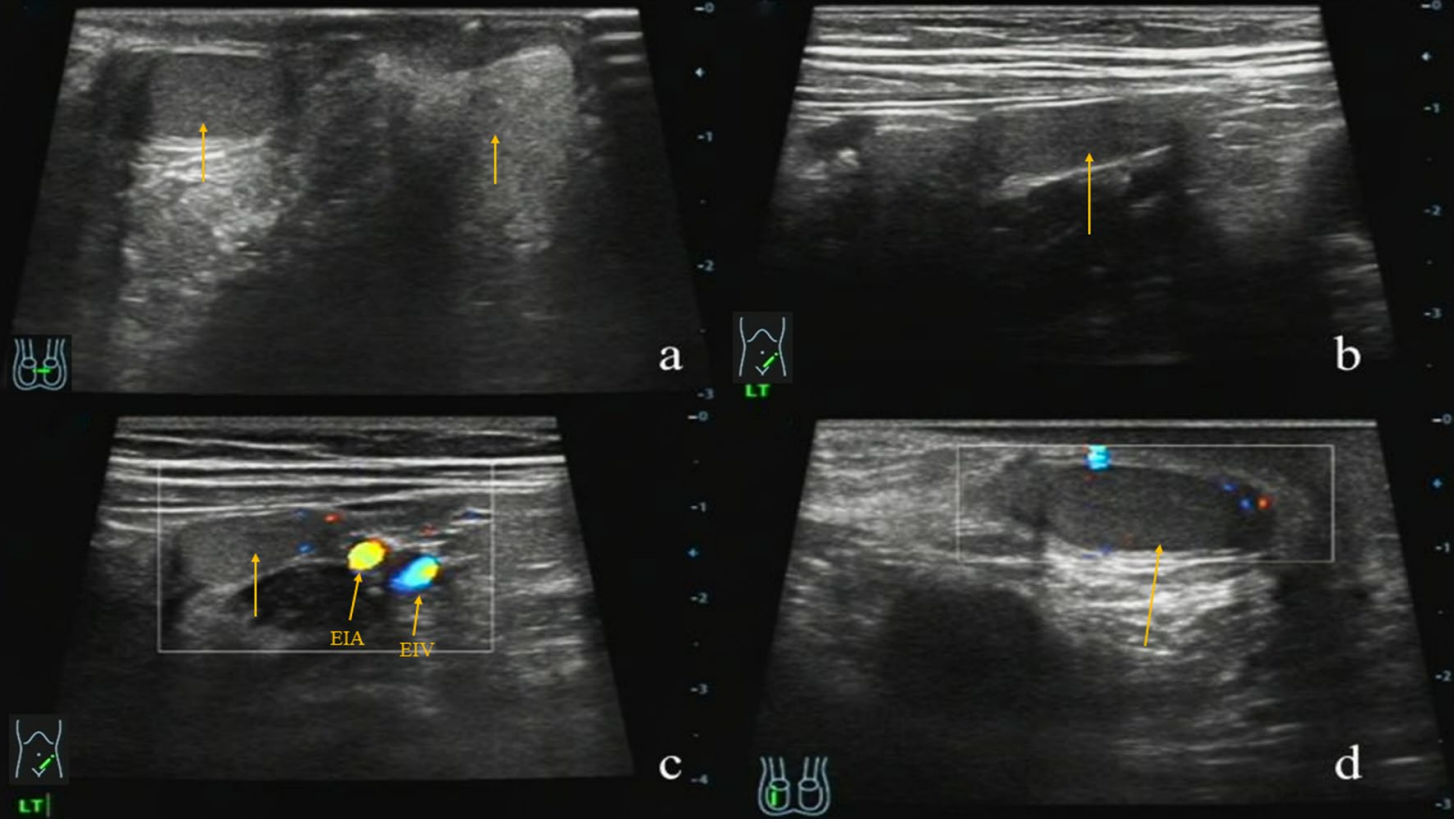
Ultrasound features of IAT on the left side of an 8-months-old boy. **(a)** The cross section of the scrotum showed that the left testicular was not in the left scrotum, and the right testicular was in the right scrotum **(b)** and the left testicular was showed next to the left external iliac artery (EIA) in the abdominal cavity **(c)** the left testis without obvious movement, CDFI showed punctate blood flow signal in the parenchyma **(d)** and the longitudinal section and the CDFI showed that the right testis was normal (arrows).

IAT and EAT sonograms showed that there were no testis and epididymis echo in the affected side’s scrotum. Testis was found in the inguinal area, the abdominal cavity, near the iliac fossa, adjacent to the iliac vessels, or other location for descending path of the testis. The testes boundary were clear and the internal parenchyma were hypoechoic. The volume were usually smaller than the healthy side testis and Color Doppler Flow Imaging (CDFI) can displayed punctate blood flow signals. The ultrasound image performance of EAT and IAT were shown in Figs. 2 and 3.

One case was misdiagnosed as IAT by preoperative ultrasound. Diagnostic laparoscopy showed that the left inner ring orifice was closed, the left spermatic cord and vas deferens were thinner than the right, and no testis was found along the left spermatic cord, and further transinguinal cryptorchid exploration was performed. It showed that the left spermatic vessel and vas deferens ended at the outer ring orifice, and the local enlargement was 4 × 3 mm nodules. The testicular nubbin and remnant cord structures were removed to confirm the diagnosis. Postoperative pathology showed that there were fibrovascular, epididymal ducts and vas deferens in the specimens, but there was no seminiferous tubules. pathologically diagnosed as testicular regression syndrome. The age, clinical manifestations and treatment of the misdiagnosed cases were shown in Table 2.

**Table 2:** Age, clinical manifestations and management of NPT cases misdiagnosed by preoperative ultrasound (10 cases in total, 12 testes).

Serial number	Age (years)	Affected side	Preoperative Ultrasound	Diagnostic laparoscopy (testis location)	Surgical procedure	Pathological diagnosis
1	1.6	L	IAT	NVT (left superficial inguinal ring, dysplastic testicular nodule of the left spermatic cord terminal)	AO	TRS (no seminiferous tubules were seen inside, but fibrovascular, epididymal ducts and vas deferens were seen inside.)
2	7.75	R	NVT	IAT (behind the ascending colon, poorly developed, without the vas deferens and testicular leads)	O	Dysplastic-like testicular changes (fibrovascular, epididymal ducts, vas deferens, seminiferous tubules, and few spermatogonia were seen inside)
3	1	R	NVT	IAT (right superficial inguinal ring, dysplastic testicular nodule of the left spermatic cord terminal)	AO	TRS (no seminiferous tubules were seen inside, but fibrovascular, epididymal ducts and vas deferens were seen inside)
4	1.25	B/L	NVT	IAT (next to bilateral EIA)	FSO	–
5	3	R	NVT	IAT (behind the transverse colon, but vascular development in the testis leading zone was acceptable)	LO	–
6	8	L	NVT	IAT (next to left EIA)	LO	–
7	1	B/L	NVT	IAT (inferior pole level of bilateral kidneys, no testicular lead, short spermatic vessels)	FSO	–
8	1.83	R	NVT	IAT (right deep inguinal ring)	LO	–
9	2.75	R	NVT	IAT (next to right EIA)	LO	–
10	7.84	L	NVT	IAT (next to left EIA)	LO	–

*L* left, *R* right, *B/L* bilateral, *IAT* intra-abdominal testis, *EAT* extra-abdominal testis, *NVT* non-viable testis, *LO* laparoscopic orchiopexy, *FSO* Fowler-Stephens orchiopexy, *AO* atrophic orchiectomy, *O* orchiectomy, *EIA* external iliac artery, *TRS* Testicular Regression Syndrome.

### Non-viable testis (NVT)

A total of 71 cases (73 testes) were diagnosed as NVT by preoperative ultrasound, there were 51 cases on the left side, 18 cases on the right side and 2 cases on both sides. There were 62 cases (62 testes) consistented as NVT by the diagnostic laparoscopy. There were 48 cases (48 testes) performed testicular nubbin and remnant cord structures resection. There were 14 cases (14 testes) only performed diagnostic laparoscopy, because there were only showed Blind-ending vas and vessels in the abdominal and no further exploration was necessary. There were 9 cases (11 testes) misdiagnosed. The preoperative ultrasound’s coincidence rate was 84.9%.

The NVT-testicular absent sonogram showed that there was no testis echo in the scrotum, inguinal area, retroperitoneum and perineum.

**Figure 4:**
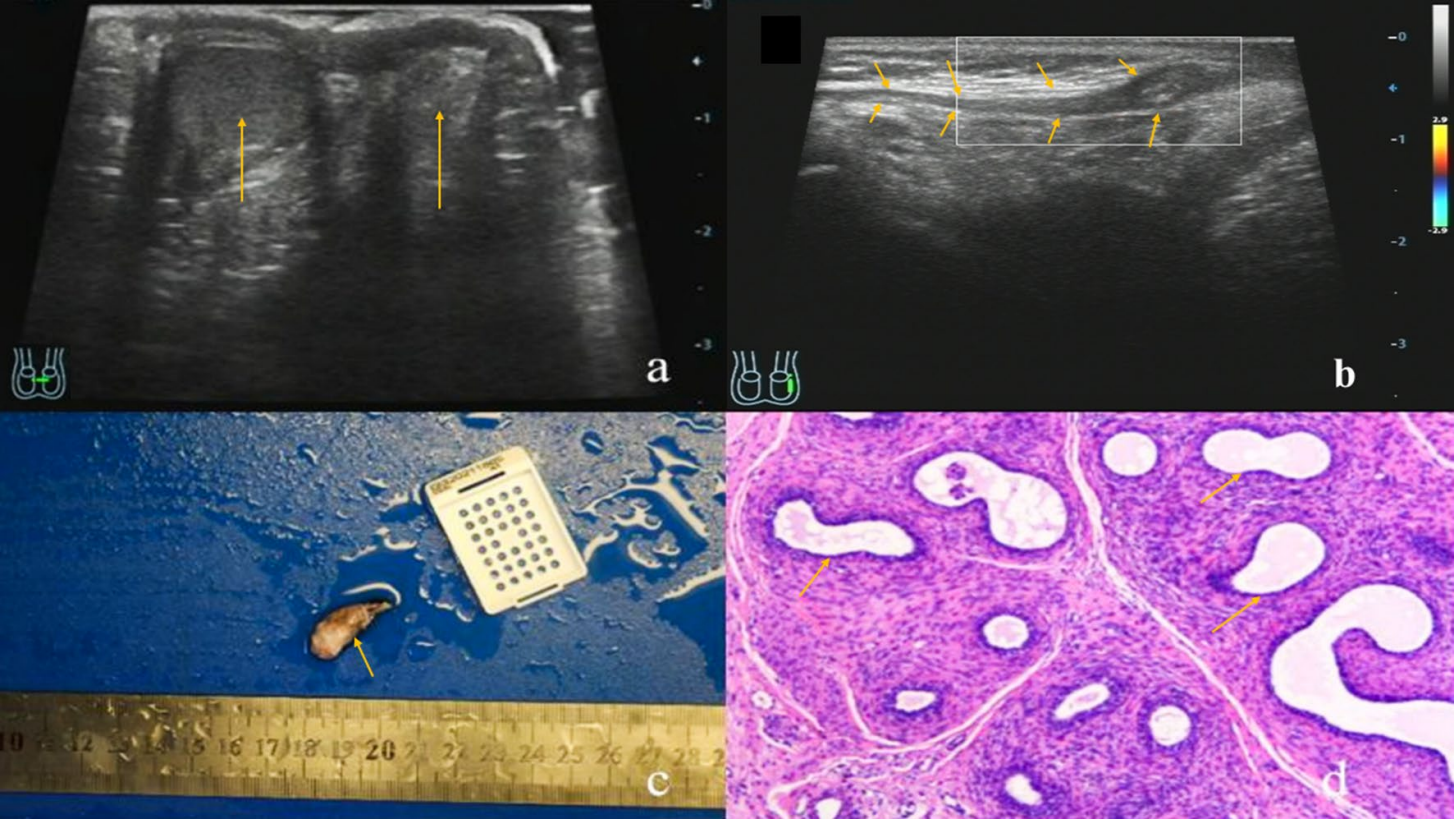
Ultrasound and pathological features of NVT on the left side in a 10-months-old boy. **(a)**The cross section of the scrotum showed that the right testis was in the right scrotum, and the left testis was not in the left scrotum **(b)** the long-axis view of the left inguinal canal showed a small hypoechoic nodule at the end of the spermatic cord on the left, with uneven internal echo. CDFI showed no blood flow signal in the nodule **(c)** the patient underwent resection of the testicular nubbin and the left spermatic cord, and the general pathology of the surgical specimen was gray-white tissue, lacking the shape and structure of the testis, and the size was 2.5 × 1.0 × 0.5 cm **(d)** under the light microscope, there were displayed fibrovascular, epididymal duct and vas deferens in the specimens, but the seminiferous ducts were not displayed and the pathological diagnosis was testicular regression syndrome (arrows).

The NVT-testicular remnant ultrasound showed that there were a small hypoechoic masses with a long strip-shaped spermatic cord in the inguinal area, the boundary were not clear, and the internal echo were not even, and the inside could show patchy hyperechoic echoes, without liquefaction. The CDFI were not showed the blood flow signal in the masses. The ultrasound image performance were shown in Fig. 4.

There were 9 cases (11 testes) misdiagnosed as NVT by preoperative ultrasound. Diagnostic laparoscopy showed that these testes were located behind the ascending colon and the front of the iliopsoas muscle at the lower pole level of the kidney, and these testes were very poorly developed. There were 5 cases (5 testes) performed laparoscopic orchidopexy, 2 cases (4 testes) performed Fowler-Stephens orchidopexy.

And the other 2 cases (2 testes) were performed orchiectomy because the diagnostic laparoscopy showed that these testes lack the structure of the vas deferens and testicular belt, which cannot meet the conditions of Fowler-Stephens orchidopexy and laparoscopic orchidopexy. These two dysplastic intra-abdominal testes were at risk of long-term malignant transformation. After the surgeon explained the condition to the parents, the parents agreed and signed an informed consent form. These two patients were performed the orchiectomy. The preoperative ultrasound were misdiagnosed NPT in 10 cases, with a total of 12 testes. The age, clinical manifestations and treatment of the misdiagnosed cases were shown in Table 2.

The SPSS Kappa consistency test was used to analyze the consistency of preoperative ultrasound and diagnostic laparoscopy in diagnosing three types of NPT (IAT, EAT, NVT). The statistical results showed that the preoperative ultrasound and diagnostic laparoscopy had good consistency in the diagnosis of NPT in this group of cases, and the Kappa value was 0.940. The crosstabulation and symmetric measures for preoperative ultrasound and diagnostic laparoscopy were shown in Tables 3 and 4.

**Table 3:** The Crosstabulation of preoperative ultrasound and diagnostic laparoscopy.

		Diagnostic laparoscopy			Total
		IAT	EAT	NVT	
**Preoperative ultrasound**	IAT	135	0	1	136
	EAT	0	103	0	103
	NVT	11	0	62	73
	**Total**	146	103	63	312

**Table 4:** The symmetric measures of preoperative ultrasound and diagnostic laparoscopy.

		Value	Asymptotic standard error^a^	Approximate T^b^	Approximate significance
**Measure of agreement**	Kappa	0.940	0.017	23.012	0.000
**N of valid cases**		312			

^a^Not assuming the null hypothesis.

^b^Using the asymptotic standard error assuming the null hypothesis.

### Pathological results

There were 51 cases performed the pathological examinations after surgery, which including 26 cases of terminal spermatic cord resection, 23 cases of atrophic orchiectomy, and 2 cases of orchiectomy. There were 5 cases showed the seminiferous tubules under the light microscope, which including 3 cases of atrophic orchiectomy and 2 case of orchiectomy. In the other 46 cases, there were only showed fibrovascular, vas deferens and epididymal ducts under the light microscope, but no seminiferous ducts can be found, the pathological diagnosed as testicular regression syndrome.

## Discussion

Generally speaking, because it is not only difficult to determine whether the testis is remnant or absent before the operation, and but also hard to location the testis’s anatomical place. There are still many controversies about the management of NPT, which is one of the challenges faced by pediatric urologists.

In the past 20 years, laparoscopy has been widely used in the diagnosis and treatment of NPT, especially IAT and NVT, and there are still controversies about the diagnosis of EAT by laparoscopy^8,9^. Laparoscopy can indicate whether the testicular vessels and vas enters the inguinal canal through the internal inguinal ring, but cannot provide information about the testis outside the abdominal cavity^10,11^.

Experienced pediatric urologists can accurately locate most of the EAT through repeated and careful palpation, also can determine the size and assess the developmental status of the testis at the same time. However, it is limited to some factors such as children's crying, obesity, testicular size, and physician’s experience, some EAT are easily misdiagnosed by palpation. The testes of IAT are mostly located next to the iliac vessels in the abdominal cavity and in the iliac fossa, which is difficult to touch on physical examination^12^.

According to the principle of as low as reasonably achievable (ALARA), the X-ray dose has a linear relationship with the corresponding biological effect, and any small dose of X-ray may lead to the occurrence of a certain biological effect. Therefore, we must pay attention to radiation-sensitive populations, which is including children and fetuses. Based on this, ultrasound has been widely used and developed in the fields of pediatrics and obstetrics^13,14^. However, compared to computed tomography (CT), magnetic resonance imaging (MRI) and other imaging modalities, real-time ultrasound has non-negligible advantages, which is low-cost, non-radiation, and high-resolution. It is not only locate the position of the testis, but also clearly show the small lesions in the inguinal canal and scrotum, as well as the abdominal and retroperitoneal lesions of infants and young children. Ultrasound can also assess the internal blood supply, size and volume of the testis. Ultrasound can real-time assess changes in testicular activity and position in children standing, supine, and crying. The presence of other comorbidities such as hernias during crying and Valsalva maneuvers can also be assessed at the same time. This is important because positioning and dynamic manipulation can affect our ability to diagnose NPT^7^.

### Ultrasonic appearance and coincidence rate analysis of IAT and EAT

Ultrasound of IAT and EAT showed that the affected scrotum were not found the echoes of testes and epididymis. The testes of IAT were located beside the iliac vessels in the abdominal cavity or near the inner ring of the iliac fossa. The testes of EAT were located at the inner ring of the inguinal canal. The volume of IAT and EAT is smaller than the healthy side, the boundary is clear, and the internal parenchyma is hypoechoic, CDFI can displayed points blood flow signals in the testis. IAT and EAT are mostly lack of mobility. In this group, there were 183 cases (239 testes) diagnosed of IAT and EAT by preoperative ultrasound, which 182 cases (238 testes) were matched with diagnostic laparoscopy. There was only one case was misdiagnosed. The positive predictive value of NPT of preoperative ultrasound was 99.58%, the sensitivity (Sn) was 95.58%.

In this group, the coincidence rate of NPT of preoperative ultrasound diagnosis was higher than other previous studies, which is related to the following factors: 1. The specific sonographic performance of cryptorchidism. IAT and EAT are hypoechoic masses outside the scrotum, with clear boundaries, which can show scattered dot-like hyperechoic calcifications in the masses, CDFI showed punctate blood flow in the masses^15,16^. Cryptorchidism is often associated with testicular microlithiasis. Ultrasonography of testicular microlithiasis is characterized by scattered dot-like hyperechoic foci in the parenchyma of the testis. Therefore, when the hypoechoic mass outside the scrotum showed scattered dots in the parenchyma on ultrasound, it is highly indicated as IAT and EAT^17–19^; 2. The average age of the children in this group was 2 years and 4 month, the abdomen was shallow, and the high-frequency probe had high resolution to the abdominal organs of infants and young children, which was conducive to the abdominal ultrasound examination of IAT and NVT; 3. In this group, the sonographers are all senior physicians with more than 10 years of clinical experience for the diagnosis and treatment of pediatric genitourinary system, which are proficient in the development and anatomical characteristics of the genitourinary system.

### Ultrasonic performance and coincidence rate analysis of NVT

NVT contains the absence of testis and remnants of testis. The sonogram of the testicular absence showed that there was no testis echo in the scrotum, inguinal canal, retroperitoneum and perineum on the affected side. The sonogram of the testicular remnant showed that there was a small hypoechoic mass at the end of the cord-like spermatic cord structure of the inguinal canal on the affected side, the boundary was unclear and the internal echo was not even, the CDFI was not showed the blood flow signal in the mass. In this group, there were 71 cases (73 testes) of NVT diagnosed by preoperative ultrasound, 62 cases (62 testes) were consistented with diagnostic laparoscopy and 9 cases (11 testes) were misdiagnosed by preoperative ultrasound. The preoperative ultrasound’s coincidence rate of NVT was 84.9%. The preoperative ultrasound’s negative predictive value (NPV) of NPT was 84.93%, the specificity (Sp) was 98.41%.

In this study, there were 9 cases (11 testes) misdiagnosed as NVT by preoperative ultrasound, which is related to the following factors: 1. Abdominal acoustic window display is limited by a lot of intestinal gas, which was difficult to distinguish blind-ending vas and other small nodules by ultrasound; 2. Ultrasound features of the inguinal testicular remnant are the small hypoechoic nodules at the end of the cord-like spermatic cord structure in the inguinal canal, and the hypoechoic nodules need to be connected to a cord-like spermatic cord structure. The points for testicular remnants by ultrasound diagnosis was to assess the anatomical relationship between the small nodules of the affected inguinal canal and the proximal spermatic cord structure. When there was a lack of obvious hypoechoic nodules at the end of the cord-like spermatic cord structure in the inguinal area, or the proximal end of the small hypoechoic mass lacked a cord-like spermatic cord structure, then there was insufficient evidence to diagnose NVT by ultrasound.

Surgical follow-up of this group of cases showed that when the NVT-testis absence of was diagnosed during the operation, diagnostic laparoscopic showed that the spermatic cord vas and vessels were blind-ending in the abdominal cavity, which was indicated a vanishing testicle. The patients only underwent diagnostic laparoscopic, and there was no further exploration is necessary (19% of cases, n = 14/71, testes = 14/73).

When the NVT-testicular remnant was diagnosed during the operation, diagnostic laparoscopic showed that the testicular vessels and vas entering the inguinal canal through the internal inguinal ring, and there was a testicular nubbin in the inguinal region or scrotum (66% of cases, n = 48/71, testes = 48/73) cases (48 testes). The patients underwent resection of the testicular nubbin and remnant cord structures on the affected side, which was good at to confirm the diagnosis and reduce the malignant transformation’s risk from testicular remnants to intratubular germ cell neoplasia (ITGCN). In this study, there were 5 cases showed the seminiferous tubules under the light microscope by the pathological examination (9.8% of cases, n = 5/51, testes = 5/51)), which was suggesting that remnant cord structures should be removed because viable residual testicular elements are present in a part of the cases. The results of this studies was consistent with previous research conclusions^20–22^.

Typically, we use Doppler ultrasonography, including color Doppler ultrasonography (CDU), power Doppler ultrasonography (PDU), and spectral pattern analysis (SMA)measurements to evaluate infants and prepubertals blood flow in the testis. The lowest pulse repetition frequency (PRF), high color gain setting, low flow filter, and low velocity scale were used to optimize detection of weak arterial flow. However, despite the combined use of CDU and PDU, blood flow signals within the testicular parenchyma before puberty may sometimes not be detected. In previous studies, the combined CDU and PDU models were used to detect normal prepubertal and postpubertal testes. Testicular parenchymal blood flow signals was detectable in only 91.7% (n = 22/24) of normal prepubertal testes, while 100% (n = 49/49) of postpubertal testis could detect testicular parenchymal blood flow signals^23^. Reasons for the low detection rate of blood flow signals in testicular parenchyma include motion artifacts when children do not cooperate, small testis size, poor contact between probe and scrotal skin, and probe stability, and different machines have different sensitivity to detecting low blood flow. We tried to overcome this by perseverance, multiple attempts, or reassurances such as giving milk and cartoons to the children during the ultrasound.

The Gel Pad is a water-based, soft, disposable pad which can improves the transmission of acoustic energy for excellent probe stability and contact, especially when scanning uneven skin surfaces such as the palm. Previous studies have shown that the application of a gel pad can significantly improve the visualization of blood flow in superficial skin areas when performing color Doppler imaging^6^. According to the previous literature, there is no large sample research report on the application of gel pads in the diagnosis of testicular ultrasound in children. It may be related to the special structure of the scrotum in the shape of an arc-shaped pouch, the position of the cryptorchidism is not fixed, the infants do not cooperate, the gel pad is difficult to place on the scrotum stably, and the lack of a stable support plane. With the advancement of ultrasound technology, the gel pad may move synchronously on the skin surface with the movement of the ultrasound probe in the future, which may increase the application of the gel pad in testicular ultrasound in children and improve the display of blood flow signals within the testicular parenchyma.

However, there are still some shortcomings in this study, which is including the number of cases of IAT is limited, and lacking of the long-term follow-up results. Especially for Fowler-Stephens orchidopexy, ultrasound and long-term testicular spermatogenesis should be followed up. Therefore, large controlled studies and long-term follow-up results are still needed for nonpalpable testis to determine the best modalities for diagnosis and treatment.

## Conclusion

Therefore, the points of NPT by preoperative ultrasound is to find IAT and EAT through standardized scanning of anatomical areas such as the inguinal canal and abdominal cavity, and further identify the possibility of the presence of NVT. Ultrasound can provide valuable information for the preoperative diagnosis of children with nonpalpable testicles, and especially good at identifying the EAT and IAT with normal testicular morphology.

## Data Availability

The datasets generated and/or analysed during the current study are not publicly available, but are available from the corresponding author on reasonable request.
